# Biological Activity of Silicon Nitride Ceramics: A Critical Review

**DOI:** 10.3390/ma17225548

**Published:** 2024-11-13

**Authors:** Francesco Boschetto, Alfredo Rondinella, Elia Marin

**Affiliations:** 1 Center for Excellence in Hip, Scottish Rite for Children, Dallas, TX 75219, USA; 2 Department Polytechnic of Engineering and Architecture, University of Udine, 33100 Udine, Italy; 3 Materials Bioengineering Laboratory, Faculty of Materials Science and Engineering, Kyoto Institute of Technology, Sakyo-ku, Matsugasaki, Kyoto 606-8585, Japan; 4 Biomedical Research Center, Kyoto Institute of Technology, Sakyo-ku, Matsugasaki, Kyoto 606-8585, Japan

**Keywords:** Si_3_N_4_, biocompatibility, silicon nitride, biological activity, history

## Abstract

The commercial use of Si_3_N_4_ ceramics in the biomedical field dates back to the early 1980s and, initially, did not show promising results, which is why their biocompatibility was not then investigated further until about 10 years later. Over the years, a change in trend has been observed; more and more studies have shown that this material could possess high biocompatibility and antibacterial properties. However, the relevant literature struggles to find mechanisms that can incontrovertibly explain the reasons behind the biological activity of Si_3_N_4_. The proposed mechanisms are often pure hypotheses or are not substantiated by comprehensive analyses. This review begins by studying the early references to the biological activity of Si_3_N_4_ and then reviews the literature regarding the bioactivity of this ceramic over time. An examination of the early insights into surface chemistry and biocompatibility lays the foundation for a detailed examination of the chemical reactions that Si_3_N_4_ undergoes in biological environments. Next, the analysis focuses on the mechanisms of bioactivity and antipathogenicity that the material exhibits both alone and in combination with modern bioglass. However, it is highlighted that despite the general consensus on the biocompatibility and bioactivity of Si_3_N_4_ ceramics, sometimes the proposed biological mechanisms behind its behavior are discordant or unsupported by the direct evaluation of specific biochemical activities. This review highlights both the reliable information in the literature and the gaps in research that need to be filled in order to fully understand the reasons behind the biological properties of this material.

## 1. Introduction

**Figure 1:**
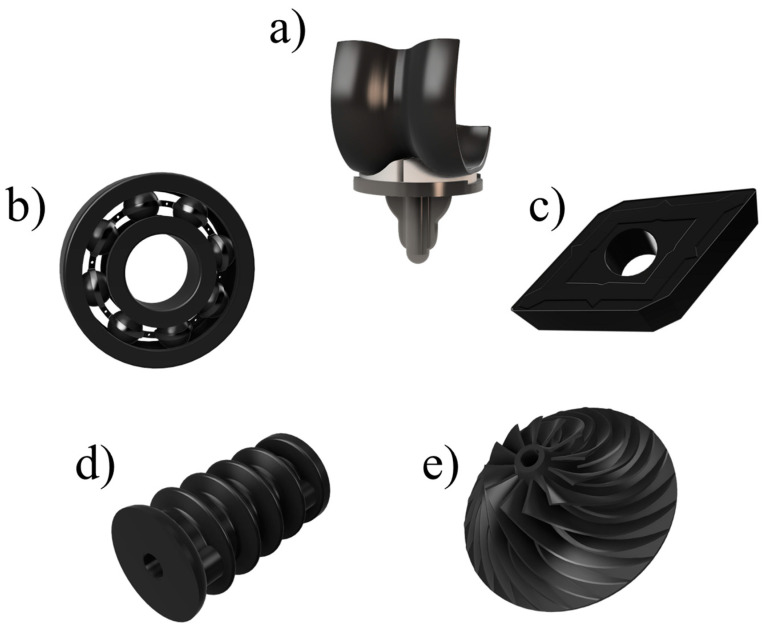
Examples of common industrial applications of Si_3_N_4_ making use of its properties: (**a**) femoral component of a knee implant (biocompatibility), (**b**) ball bearing (wear resistance), (**c**) lathe insert (hardness), (**d**) insulator (electrical resistance), (**e**) turbine pump impeller (thermal resistance).

Silicon nitride ceramics (Si_3_N_4_) are recognized for their characteristics like strong durability and resistance to wear which make them suitable for use in different areas such as in the manufacturing of cutting tools, in industrial components, and in electronics and engine components where their high hardness, thermal stability, and electrical properties provide significant advantages over traditional materials (Figure 1). Beyond these applications, however, they have received a lot of interest also in the biomedical field lately as they transitioned from being used in high tech industries to becoming promising materials, for healthcare applications as well. Their ability to withstand harsh conditions while remaining compatible with the body makes them a favorable choice for various medical settings requiring both toughness and biocompatibility.

Silicon nitride stands out among ceramics not for its mechanical strength but also for its fascinating biological properties that researchers have discovered to be beneficial, for supporting the vital cellular functions necessary for seamless integration into biological environments. Studies indicate that silicon nitride exhibits interactions with cell types such as osteoblasts by encouraging their attachment and proliferation. This dynamic relationship plays a role in enhancing the material’s capacity to fuse with neighboring tissues and foster the processes of healing and regeneration.

Nevertheless, it is worth noting that while there have been discoveries in the field of biomaterials related to silicon nitride material properties and their effects, there is still a lack of understanding regarding the underlying biological mechanisms at work. The outcomes of the various studies conducted on this matter are different. These inconsistencies pose inquiries into the impact of manufacturing techniques and material characteristics on functionality. 

This review aims, by synthesizing the findings from a broad spectrum of studies, to provide a comprehensive evaluation of the current state of knowledge regarding the biological activity of silicon nitride ceramics; with a focus on critically analyzing the gaps in the current research, we seek to clarify the role of silicon nitride in biomedical contexts and highlight the areas for further understanding its place in medicine.

### Chronological Development of Biological Perspectives on Silicon Nitride Ceramics

**Figure 2:**
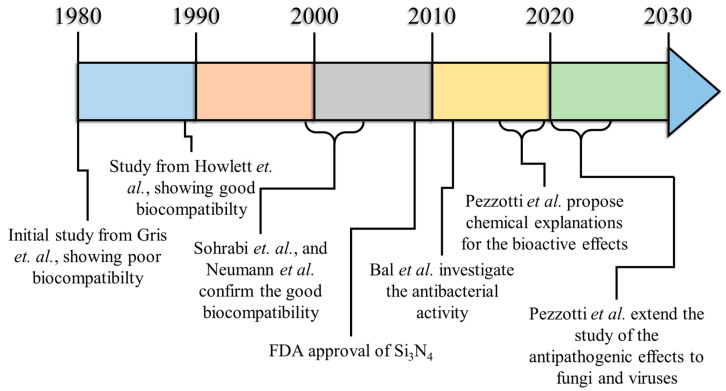
Research timeline: the evolution of Si_3_N_4_ as a biomaterial.

The first published research involving the biocompatibility testing of Si_3_N_4_ ceramics dates back to 1980 (Figure 2, Table 1), when Griss et al. published “Alumina, ceramic, bioglass and silicon nitride: a comparative biocompatibility study” showing that, overall, it was the least compatible material when compared to stainless steel, Al_2_O_3_, and bioglass [1]. After Griss, only a few other research articles from the 1980s mention the use of Si_3_N_4_ as a structural biomaterial. In the two volumes of *Metal and Ceramic Biomaterials*, Vol. I, *Structure* and Vol. II, *Strength and Surface*, published in 1984 by CRC Press (Taylor and Francis Group) [2,3], silicon nitride is already mentioned as a biomedical material with outstanding mechanical performances, but its actual biocompatibility was not further questioned. The idea behind the applicability of Si_3_N_4_, at the time, was probably based on the misconception that it could be completely bio inert because of its intrinsic chemical resistance at high temperatures and in aggressive media. This idea is based on the scarce biomedical literature of the time: silicon nitride has been used as an insulating layer to protect various biomedical devices, in particular electrodes [4,5,6,7], and no mention of its bioactivity can be found in the scientific literature before 1989.

In the pioneering article, “The effect of silicon nitride ceramic on Rabbit Skeletal Cells and Tissue—an in vitro and in vivo investigation” [8], Howlett et al. presented a morphological assessment of the effect of silicon nitride ceramic (Si_3_N_4_) on rabbit marrow stromal cells and their differentiation when grown in vitro and in vivo. According to the authors, Fresh marrow and marrow stromal cells formed bone, cartilage and fibrous tissue in contact with Si_3_N_4_. Moreover, Si_3_N_4_ devices implanted in adult rabbits were enclosed by a stable cuff of bone within four months of implantation and remained unchanged during the rest of the animal’s life. Despite the authors concluding that “Si_3_N_4_ has the potential of an important ceramic for use in osseous reconstruction”, the research in this field would proceed slowly and without continuity for more than two decades.

In a letter to the editor of *Clinical Orthopaedics and Related Research* [9], Prof. Peter Griss (author of the paper from 1980), raised concerns about Si_3_N_4_’s actual biocompatibility, claiming that Howlett et al. tested the ceramic without a comparative material of established good or bad biocompatibility, which considerably reduces the validity of the conclusions drawn from the experiments. Moreover, in the letter, Griss reports that in a new femur implantation model, Si_3_N_4_ shows a similar lack of biocompatibility. Griss concludes the letter stating that Si_3_N_4_ should not be recommended as a candidate endoprosthesis material, but that more experimental quantitative data were needed for a final statement.

The results of the femur implantation model of prof. Griss would be ultimately published in 1992 [10]: again, Si_3_N_4_ (as well as SiC) resulted in being less biocompatible than the reference material (alumina).

A few years later, Sohrabi et al. studied the pro-inflammatory cytokine expression by human osteoblast-like cells upon their exposure to silicon nitride. The results showed that Si_3_N_4_ is biocompatible, and its exposure to a wide range of silicon nitride particle concentrations (1, 10, 100mg/mL) does not hinder the proliferation or induce pro-inflammatory cytokine expression of human osteoblast-like cells [11]. In the same year, the same research group also found that silicon nitride stimulates the proliferation and the osteocalcin production of human osteoblast-like cells [12], further supporting the hypothesis that Si_3_N_4_ is biocompatible and bioactive, but again without providing a direct comparison with an adequate reference material, such as alumina.

While the in vivo results from Griss showed an overall inadequate biocompatibility of Si_3_N_4_ in vivo, similar experiments conducted by Neumann et al. [13] showed good osseointegration and a lack of immuno-inflammatory reactions, suggesting that Si_3_N_4_ has similar biocompatibility features as the Al_2_O_3_ control. Neumann et al. further noted that “During the period of 4 to 8 weeks after implantation, there was a significant decrease of bone-implant attachment for Al_2_O_3_ compared to Si_3_N_4_” [14].

In a later attempt to extinguish the existing controversy between Griss and Howlett, Neumann et al. performed a series of comparisons of silicon nitride and alumina samples with different chemical compositions [15]. All the silicon nitride and alumina samples resulted in being biocompatible in vitro, with no sign of cytotoxicity. Neumann further noted that “The comparison of cell counts with the distribution of elements in the different silicon nitride samples shows no correlation”, but it is important to note that all the Si_3_N_4_ specimens used by Neumann had overall similar chemical compositions, with Mg between 0.1% and 0.5%, Al between 1.1 and 2.4%, and Y between 0.9 and 1.5%. Considering the innate variability of the in vitro assessments, the effects of these elements could have been well below the detection limit for the method used. Neumann et al. also tested a prototype of a silicon nitride mini-plate osteofixation system for the midface [16], observing satisfactory results in terms of intraoperative handling, mechanical stability, and biocompatibility, in particular when compared to Al_2_O_3_. Similar results were also obtained by Ruan et al., working on endosteal implants in dogs [41].

A more recent, in vitro comparison of oxide and non-oxide materials was published by Cappi et al. in 2009 [20]. In their experiments, cold isostatic pressed and hot isostatic pressed Si_3_N_4_ were compared to Al_2_O_3_, ZrO_2_, and SiC. The study came to the conclusion that the non-oxide ceramic materials, Si_3_N_4_ and SiC, either produced via pressureless sintering or hot-pressing, are cytocompatible for human mesenchymal stem cells, and allow for osteogenic differentiation.

In the early 2000s, further insights into the potential bioactivity of Si_3_N_4_ emerged also from the field of microelectromechanical systems: in an in vitro test using platelet rich-plasma, the authors observed that thin films of Si_3_N_4_, Si_1.0_N_1.1_, and Si cause more platelet adhesion when compared to SiO_2_, increasing the risk of thrombogenesis [42]. Reducing the risk required a drastic modification of the surface chemistry of Si_3_N_4_, for example going towards silicon oxynitride films [21]. The further functionalization of the Si_3_N_4_ thin film surfaces, for example with primary amine functional groups (NH_2_), stimulated the adhesion and spreading of an osteoblast-like cell line [22] but, remarkably, the highest spontaneous alkaline phosphatase activity was observed for cells grown on un-functionalized Si_3_N_4_ substrates.

Another common method to estimate the cytotoxicity of a material is to use particulates. Unlike bulk materials, particulates offer a higher surface-to-volume ratio, strongly exacerbating chemical and biological reactions. Furthermore, particulate cytotoxicity is also strongly affected by the average particle diameter (nanometric particles can penetrate cellular membranes) and shape (particles with sharp edges are usually more cytotoxic than round particles. The earliest assessment of Si_3_N_4_ particulate cytotoxicity can be found in a 2003 paper from Yamamoto et al. [23], for L929 and J744A.1 cell lines. As observed before by Neumann et al. for bulk ceramic discs, the behavior of Si_3_N_4_ resulted in being similar to that of Al_2_O_3_ and, overall, more cytotoxic at lower concentrations with respect to TiO_2_ and SiC.

In 2006, new research from Bogner et al. on biocompatible microelectronic materials tested on Caco-2 cells partially contradicted previous results, showing that in p-Si wafers coated with Si_3_N_4_ (as well as Au, Al, and ITO), the alkaline phosphatase activities only reached between 15% and 35% [24].

By 2008, Bal et al. were testing silicon nitride ceramic bearings for total hip arthroplasty [17,18,19]. The components, obtained via sintering and HIPping, showed superior fracture toughness and flexural strength than the reference Al_2_O_3_, and did not change their properties after autoclaving. In another paper from 2012, Bal et al. [43] noted that Si_3_N_4_ could be a superior competitor to the more common Al_2_O_3_-based orthopedic ceramics on the market (Zirconia-toughened alumina), a prediction that, at least from the tribological point of view, has been contested by other researchers [44]. 

Titanium and PEEK, both possessing hydrophobic characteristics with net negative surface charges, were compared to Si_3_N_4_, which exhibited a hydrophilic surface with a net positive charge. Notably, two surface finishes of Si_3_N_4_ were explored: as-fired and polished. The study revealed a decreased biofilm formation and fewer live bacteria on both the as-fired and polished Si_3_N_4_ surfaces compared to Ti and PEEK. These observations suggested that the differential surface chemistry and nanostructured topography of Si_3_N_4_ played a pivotal role in impeding bacterial biofilm formation, colonization, and growth.

The investigation extended beyond the bacterial response to examine protein adsorption on material surfaces, specifically focusing on fibronectin, vitronectin, and laminin. Remarkably, Si_3_N_4_ demonstrated significantly greater amounts of these proteins adhering to its surface compared to Ti or PEEK. This finding highlighted the influence of the surface properties on the adsorption of physiologic proteins, suggesting a potential correlation with the observed in-vitro differences in the bacterial affinity for the respective biomaterials.

The findings of this study propose a novel strategy in designing future orthopedic implants based on the intrinsic biomaterial properties. The hydrophilic nature, positive surface charge, and enhanced protein adsorption on Si_3_N_4_ present an encouraging prospect for developing implants with inherent resistance to bacterial colonization. This paradigm shifts the open avenues for advancing orthopedic implant technology by exploiting the surface properties of biomaterials to mitigate the risks associated with bacterial infections. The differential responses observed in bacterial affinity and protein adsorption suggest that the unique surface properties of Si_3_N_4_ hold promise for the development of orthopedic implants with enhanced resistance to bacterial colonization.

Following the groundbreaking results of the 2012 in vitro study on the biological activity of silicon nitride (S_i3_N_4_) in orthopedic implants, the subsequent research endeavors sought to validate these findings through in vivo testing [26]. In the same year, a pivotal study investigated the performance of Si_3_N_4_ implants in comparison to poly(ether ether ketone) (PEEK) and titanium (Ti) implants [45], emphasizing the critical aspects of bone formation and bacterial resistance in orthopedic applications.

Dense implants made of Si_3_N_4_, PEEK, or Ti were surgically implanted into rat calvarial defects to simulate real-world orthopedic scenarios. Bacterial infection was induced by injecting Staphylococcus epidermidis, and control animals received saline only. The rats were sacrificed at intervals of 3, 7, and 14 days post-surgery, as well as at 3 months, to examine new bone formation and the presence or absence of bacteria.

Three months after surgery in the absence of bacterial injection, Si_3_N_4_ demonstrated an impressive ∼69% new bone formation around the implant, outperforming both PEEK (24%) and Ti (36%). In the presence of bacteria, Si_3_N_4_ maintained superior performance with new bone formation at 41%, compared to 26% for Ti and 21% for PEEK. Notably, live bacteria were identified around the PEEK (88%) and Ti (21%) implants, while none were present adjacent to Si_3_N_4_.

Despite not being the first published articles to demonstrate the bioactivity of Si_3_N_4_, the in vitro and in vivo studies of 2012 provided compelling evidence supporting the favorable attributes of Si_3_N_4_ orthopedic implants and can be considered as the starting point for the industrial interest for bioactive silicon nitride ceramics. The superior new bone formation and resistance to bacterial infection observed for Si_3_N_4_, as compared to Ti and PEEK, underscore the potential clinical significance of Si_3_N_4_ as a biomaterial for orthopedic implants.

However, as can be seen from Table 1 and Table 2, which list the most significant works regarding the biocompatibility and antipathogenic activity of Si_3_N_4_, respectively, there is no unified consensus on the mechanisms related to bioactivity.

## 2. Early Notes on Surface Chemistry and Biocompatibility

It is important to note that, up to 2012, all the investigations into the biocompatibility of Si_3_N_4_ did not provide meaningful insights about its chemical structure, to the point that most research articles do not even specify the chemical composition of the ceramic utilized.

A paper from Bock et al. [57], dating back to 2015, is arguably the first piece of scientific literature to properly investigate the chemical structure of the surface of as-fabricated and polished silicon nitride subjected to different post treatments. The authors found that both the surface chemistry and morphology of Si_3_N_4_ can be varied through conventional thermal, chemical, and mechanical treatments, with as-fabricated Si_3_N_4_ exhibiting anisotropic grains covered with a thin Si_2_N_2_O passivation layer, strongly negative charging at biological pH, and moderate hydrophilicity. Etching in HF produced a surface composition with a higher N/Si and lower O/Si ratios than as-fabricated materials, strong negative surface charging at homeostatic pH, and moderate hydrophilicity. Thermal treatment in N_2_ produced a surface coated in crystalline β-Si(Y)AlON precipitates and exhibited extreme hydrophilicity. Thermal treatment in an oxidizing atmosphere resulted in a surface composition effectively comparable to amorphous SiO_2_. It also exhibited extremely low wetting angles and charging behavior that mimicked pure silica [58]. The isoelectric points of these variously treated samples increased with decreasing O/Si and with increasing N/Si atomic ratio, as the surfaces transitioned from resembling pure SiO_2_ to pure Si_3_N_4_.

When tested with human osteosarcoma cells (SaOS-2), the same surface treatments resulted in marked differences in the amounts of bone tissue produced, with the N_2_-annealed specimen reaching about 40% higher bone tissue volumes per unit of area. The author concluded that “alterations of its lattice defects promote proliferation of osteoblasts and the subsequent generation of natural hydroxyapatite. Surface modulation of Si_3_N_4_ illustrates the concept that engineering of atomically defective biomaterials can lead to remarkable osteoconductive characteristics”. A more detailed explanation was published in a follow-up paper [27], where the authors claimed that the peculiar SiYAlON phase on the surface of these specimens contained an abundance of positively charged N-vacancies and N–N bonds which modulated the negative surface charge developed by the deprotonation of amphoteric silanols. The various hypotheses for the improved bioactivity of Si_3_N_4_ will be discussed in detail in the following section, but this paper represents the first attempt to rationalize the biological behavior of silicon nitride from a chemical point of view.

The same research group tested surface-modulated Si_3_N_4_ samples against Porphyromonas gingivalis [46], showing that the oxidized (hydroxyl-rich) Si_3_N_4_ was less effective in inducing PG lysis when compared to as-sintered and HF-etched (amine-rich) Si_3_N_4_. The N/Si (O/Si) ratios were 1.22 (0.17), 1.05 (0.15), and 0.09 (1.98) for the as-sintered (polished), HF-etched, and oxidized samples, respectively. Unfortunately, the authors did not provide any quantitative analysis, but the fluorescence imaging shows a weak green CFDA signal for the oxidized surface, which the authors interpreted as a sign of weaker bacterial lysis.

## 3. Crystal Structure of Biomedical Si_3_N_4_

**Figure 3:**
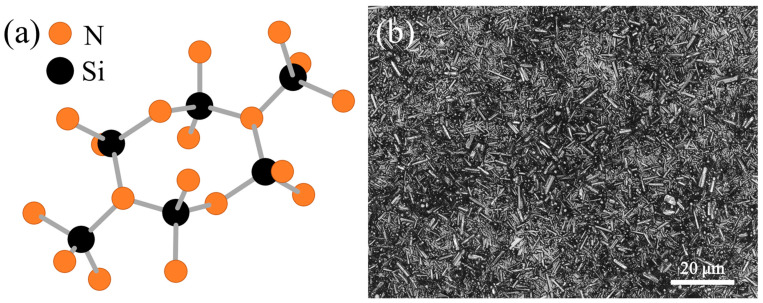
(**a**) Structure of β-Si_3_N_4_ and (**b**) typical microstructure of an as-sintered β-Si_3_N_4_ specimen, as observed at 150×.

Silicon nitride (Si_3_N_4_) exists in two primary crystalline forms, alpha (α) and beta (β), which differ in their atomic arrangements and, consequently, their properties. The α-phase has a hexagonal structure that incorporates stacking faults, resulting in lower thermodynamic stability, while the β-phase adopts a more stable hexagonal lattice (Figure 3a) with elongated grains that interlock, conferring superior mechanical properties such as toughness and fracture resistance. In biomedical applications, β-Si_3_N_4_ is almost exclusively used due to these characteristics, which are essential for load-bearing implants and other medical devices where structural integrity and durability are critical. Additionally, the β-phase has been reported to exhibit favorable biocompatibility and bioactivity, promoting bone growth and integration, making it the preferred choice in regenerative and orthopedic applications. 

The microstructure of β-Si_3_N_4_ is characterized by the presence of elongated, acicular (needle-like) grains that interlock to form a tough, resilient network (Figure 3b). These grains play a crucial role in enhancing the material’s toughness: they act as crack-bridging elements, forcing cracks to deflect or deviate around them rather than following a straight path. This crack deflection mechanism significantly increases the energy required for crack propagation, imparting exceptional fracture toughness to β-Si_3_N_4_. To achieve this structure during sintering, specific additives—typically oxides like yttria (Y_2_O_3_) and alumina (Al_2_O_3_)—are essential [59]. These additives promote liquid-phase sintering by lowering the temperature required for densification, enabling the growth and bonding of β-phase grains into a strong, interlocking microstructure.

Interestingly, early investigations on α- and β-Si_3_N_4_-reinforced PEEK showed that, despite the lower stability of α-Si_3_N_4_, its antibacterial effect is negligible, even though its biocompatibility is comparable to, if not slightly superior to, that of β-Si_3_N_4_ [60].

## 4. Chemical Reactions of Si_3_N_4_ In Vivo

**Figure 4:**
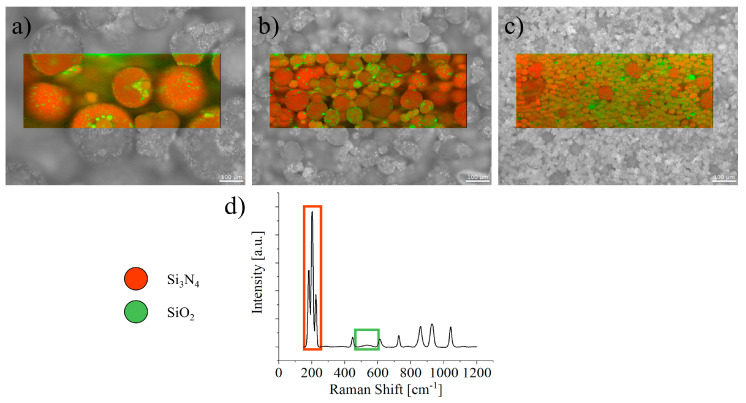
SiO_2_ and Si_3_N_4_ surface coverage on Si_3_N_4_ particles of different nominal sizes, (**a**) 300, (**b**) 50 and (**c**) 20 μm, as measured by Raman spectroscopy using the bands in (**d**) [61].

Silicon nitride (Si_3_N_4_) naturally undergoes surface oxidation when exposed to humid environments, forming a thin silicon oxide layer that plays a crucial role in its stability and activity. This oxidation effect is particularly pronounced for Si_3_N_4_ particles, with an increasingly significant impact as the particle size decreases. In smaller particles, the larger surface-area-to-volume ratio enhances the exposure to atmospheric moisture, leading to accelerated oxidation (Figure 4). This intensified oxidation in fine particles can influence their reactivity, making them more susceptible to environmental changes and potentially affecting their performance in applications such as biomedical implants or wear-resistant coatings.

Low-temperature dissolution of Si_3_N_4_ particulates has been described in detail multiple times. The hydrolysis of silicon nitride is often described by the simplified reaction:

$$Si_{3}N_{4}+6H_{2}O↔3SiO_{2}+4NH_{3}$$

assuming that silica is the main hydrolysis product. Considering that Si_3_N is the highest species formed on the surface of Si_3_N_4_, we can instead expect the following hydrolysis reactions [62]:

$$\begin{array}{c} Si_{3}N+3H_{2}O↔3SiOH+NH_{3}\\ Si_{3}N+H_{2}O↔Si_{2}NH+SiOH\\ Si_{2}NH+H_{2}O↔SiNH_{2}+SiOH\\ SiNH_{2}+H_{2}O↔SiOH+NH_{3} \end{array}$$

And we know from previous studies that silanol (Si-OH) is the major surface group on silica, while on silicon nitride, the major surface groups are amine (Si_2_-NH) and silanol (Si-OH), which are produced by a spontaneous oxidation of silicon nitride [63]. These surface groups can acquire a charge in aqueous solution according to the following reactions. The zeta potential of silicon nitride is higher due to the positively charged amine groups on the silicon nitride surface:

$$\begin{array}{c} Si-OH↔SiO^{-}+H^{+}\\ Si_{2}NH+H^{+}↔Si_{2}NH_{2}^{+} \end{array}$$

Which are responsible for the amphoteric behavior of the Si_3_N_4_ surface, as previously reported [64]. FTIR analysis performed on Si_3_N_4_ powder evidenced the presence of –NH_2_ surface groups, further supporting this hypothesis [65,66]. Additional studies on silicon nitride powder have also shown that the oxidized surface layer dissolves in the same fashion as silica [67,68]:

$$SiO_{2}+2H_{2}O↔Si{\left(OH\right)}_{4}$$

While, depending on the pH of the environment, ammonia can be converted to ammonium ions and vice versa [69]:

$$NH_{3}+H_{3}O^{+}↔NH_{4}^{+}+H_{2}O$$

The total ammonia ($T_{amm}$) is the sum of $\left[NH_{3}\right]$ + $\left[NH_{4}^{+}\right]$ and the pK of this ammonia/ammonium ion reaction is around 9.5. The amounts of each of the two species can be calculated from the Henderson–Hasselbalch equation if the pH and appropriate pK are known [69]:

$$NH_{4}^{+}=\frac{T_{amm}}{1+\log^{-1}\left(pH-pK\right)}=T_{amm}-NH_{3}$$

Molecular dynamics simulations also suggest that hydrolysis may proceed through the nucleophilic attack of water with the formation of an intermediate molecular complex involving a penta-coordinated silicon [11,12].

One last chemical compound that has been associated with the dissolution of Si_3_N_4_ in biological environments is peroxynitrite ($ONOO^{-}$), a reactive nitrogen species usually formed when nitric oxide (NO) reacts with the superoxide anion ($O_{2}^{-}$). It was first identified as a mediator of cell death in animals but was later shown to act as a positive regulator of cell signaling, mainly through the posttranslational modification of proteins by tyrosine nitration [70]. The first (and only) experimental observation of the formation of peroxynitrite on Si_3_N_4_ was performed by Pezzotti et al. using Raman spectroscopy [46], but without providing any further validation using a consolidated detection method. The band was reported to be located at 1044 cm^−1^, and can be easily confused with one of the bands of β-Si_3_N_4_ (1040 cm^−1^). Moreover, careful observations of the spectra provided confirmed that the actual band is located at 1032 cm^−1^, casting further doubts on the original observation and weakening the hypothesis of peroxynitrite formation on Si_3_N_4_. Nevertheless, the claim has been subsequently referenced various times in the literature, without providing any additional supporting evidence [47,48,49,50,71,72].

Despite the apparently simple surface chemistry, the dissolution of Si_3_N_4_ can proceed at different paces and follow different paths depending on parameters such as pH, oxidation status (in particular the ratio between Si-OH and Si_2_-NH), specific surface area and chemical composition, as evidenced by the variations in the isoelectric point reported in the literature [57,73,74,75]. The complexity of these interactions, and the lack of a comprehensive prediction model might be responsible for the variability in the biological responses observed, in particular in the early literature.

## 5. Bioactive Mechanisms

Multiple literature references support the hypothesis that Si_3_N_4_ (as well as Si and SiO_2_) is not cytotoxic [28,29] unless in the form of nano-particulates [30], in which case it might induce changes in the genes related to apoptosis, DNA damage or repair, and oxidative stress. It should be observed that, in many cases, when tested in vitro, Si_3_N_4_ performed as well as the bio-inert controls, but did not exhibit an enhanced cellular proliferation [11,76].

**Figure 5:**
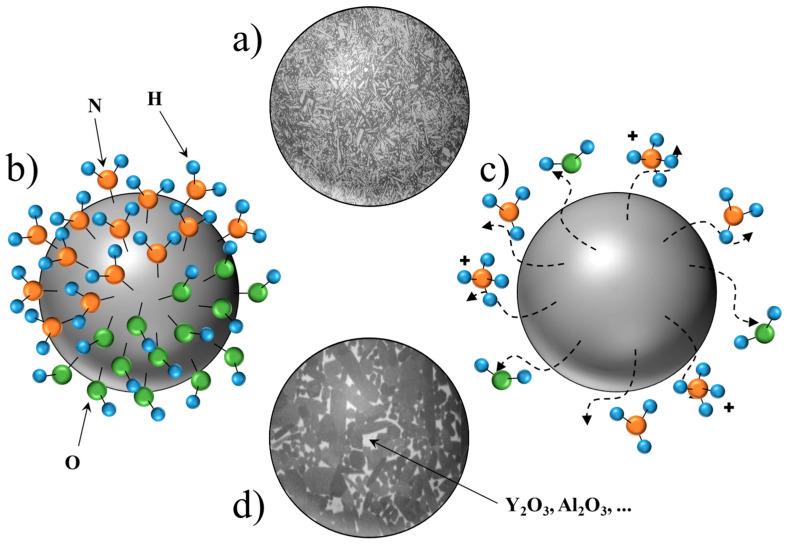
The four chemical explanations for the bioactive behavior of Si_3_N_4_, according to the literature: (**a**) surface roughness, (**b**) functional groups on the surface, (**c**) release of bioactive species, and (**d**) presence of bioactive additives. Dashed arrows indicate release of molecules from the particle, solid lines indicate the specific ion/molecule. A “+” sign indicates that the molecule is in ionic form.

Despite the numerous proposals from researchers, the exact origin of the bioactivity of silicon nitride (Si_3_N_4_) remains unconfirmed, leading to an ongoing debate in the scientific community. The hypotheses surrounding this phenomenon can be categorized into four main areas: the first focuses on the effect of surface roughness (Figure 5a), suggesting that the variations in texture may enhance cellular adhesion and proliferation. The second category examines the influence of the surface functional groups (Figure 5b), positing that specific chemical functionalities on the Si_3_N_4_ surface may play a crucial role in mediating the interactions with biological tissues. The third hypothesis revolves around the release of bioactive species (Figure 5c), proposing that the ions or compounds leaching from Si_3_N_4_ could contribute to its bioactive properties. Finally, the fourth category considers the role of bioactive additives (Figure 5d), where the incorporation of specific materials may augment the bioactivity of Si_3_N_4_ in biomedical applications.

According to Pezzotti et al., both Si and N available at the interface with Si_3_N_4_ are associated with the upregulation of the metabolic activity of osteoblasts [31], which in turn results in rapid and efficient bone growth. In preliminary research from the same authors, the amount of hydroxyapatite (mineralized bone) formed is higher for samples containing extra nitrogen [27], a result that, apparently, contradicts the observations made regarding Si- and N-rich PVD coatings [32], where N-rich Si_3_N_4_ showed higher cellular proliferation and better antimicrobial effects, while Si-rich Si_3_N_4_ showed higher amounts of bone tissue formed. Further investigations of Si-rich laser-cladded coatings of Si_3_N_4_, showed results comparable to the titanium control, but superior to both zirconia and polyethylene [33]. In yet another manuscript from the same research group, the authors noted that both cellular proliferation and bone growth diminish with decreasing nitrogen content in Si_3_N_4_ [34].

The nitrogen released from the surface of Si_3_N_4_ takes the form of ammonia or ammonium ions, depending on the environmental pH. In normal cellular metabolism, free ammonium ions are produced and consumed regularly. Glutamine synthetase utilizes free ammonium ions to produce glutamine in the cytosol, whereas glutaminase and glutamate dehydrogenase generate free ammonium ions in the mitochondria from glutamine and glutamate, respectively [77]. Ammonia and bicarbonate are condensed in the liver mitochondria to yield carbamoylphosphate, initiating the urea cycle, the major mechanism of ammonium removal in humans [77]. Martinelle and Häggström [78] observed that there is a clear difference between the ammonia/ammonium that is added to the cells and which is formed by the cells during the metabolism of amino acids, especially glutamine and glutamate, with the former leading to predictable intracellular and extracellular pH (pHe) changes. The authors postulated that one important toxic effect of ammonia/ammonium is an increased demand for maintenance energy, caused by the need to maintain the ion gradients over the cytoplasmic membrane. Other researchers found that ammonia induces the overproduction of ROS, decreases MMP, interrupts Ca^2+^ homeostasis, and subsequently causes cell apoptosis, via the P53-BAX-BCL2 and mitochrondial apoptotic pathways [79,80].

The previous literature on the bone tissue formed on Si_3_N_4_ biomaterials hypothesized that nitrogen can be substituted to oxygen in both the (PO_4_)^3−^ of and (SiO_4_)^4−^ tetrahedra and the OH group as well, but the hypothesis is not supported by the XPS analysis provided by the authors, as no additional bond, such as Ca–N, are shown to have formed, either from the Ca or the N binding energy intervals [31].

Based on the previous literature references, there seems not to be enough strong evidence to support the hypothesis that the nitrogen released from the surface of Si_3_N_4_ can have a beneficial effect on the cellular proliferation and/or metabolism, but this does not mean that nitrogen makes no positive contribution to the biocompatibility of Si_3_N_4_. As previously observed, in aqueous environments, the surface of Si_3_N_4_ is mainly composed of two species, hydroxides and secondary amines. Of these two structures, the secondary amines are more likely to exhibit bioactive properties, in particular towards collagen [81], resulting in an increased cellular adhesion [82,83]. Moreover, NH_2_ functionalized bioglasses were also reported to increase cellular proliferation, when tested with human bone marrow-derived mesenchymal stem cells [84]

Nitrogen elution from Si_3_N_4_ is regulated by the environmental pH, specific surface area, and surface chemistry. Compared to the “friendly nitrogen kinetics” hypothesis [71], which would require a case-by-case material design optimization to avoid potential cytotoxic effects, the “bioactive surface amines” hypothesis finds more support in the literature, as bioactivity was observed in a wide range of Si_3_N_4_ compositions, surface morphologies, and experimental settings. Despite the claims, no experimental proof has been provided so far for the positive effects of nitrogen elution from Si_3_N_4_.

Apart from surface amines, the chemical composition of the ceramic is also likely to play a crucial role in the biocompatibility, in particular considering the results from the early testing on Si_3_N_4_ ceramics with low amounts of additives, such as the ones reported by Griss [9,10]. Most of the Si_3_N_4_ materials that are used nowadays for biomedical applications contain a not negligible amount of Y_2_O_3_, used as a sintering aid. The recent literature results showed that Y_2_O_3_ increases the cellular proliferation and bone tissue formation in vitro, even if in very low concentrations [85,86]. Honma et al. performed a direct comparison between Si_3_N_4_ and Y_2_O_3_ powders, showing that when the two materials are mixed together, osteoblasts preferentially grow on Y_2_O_3_, where they also produce higher amounts of mineralized bone tissue [87]. The authors hypothesized that this could be due to the antioxidant action of Y_2_O_3_, while Xiang et al. observed that Y_2_O_3_ particles contribute to the inhibition of the ROS/NF-κB pathway, which plays a role in the immune response, and cell proliferation and differentiation [88]. The absence of Y_2_O_3_ might also explain why Griss et al. reported such poor biological performances for Si_3_N_4_ [9,10] when compared to subsequent research [10,89]. A correlation between the presence of Y and the bioactivity of ceramics would also explain the in vitro results obtained for the SiYAlON phase by various independent research groups [27,90,91].

Other additives in Si_3_N_4_ ceramics, such as CaO and Ca_3_(PO_4_)_2_, have been shown to influence both structural and biological properties, supporting the hypothesis of a significant contribution by these additives to bioactivity. Studies have demonstrated that such additives can enhance the bioactivity by creating glassy intergranular phases and promoting surface porosity. For example, flame-treated Si_3_N_4_ ceramics incorporating Ca_3_(PO_4_)_2_ exhibit increased cell viability and bone integration by forming bioactive layers rich in hydroxyapatite and calcium silicate phases, which favor cellular adhesion and proliferation [92]. Moreover, silicon-rich compositions enhance the bone mineralization, suggesting a synergistic effect of composition and structure [32]. Apart from calcium-based compounds, the addition of other oxides such as SrO, MgO, and SiO_2_ improved the cellular proliferation and differentiation [93]. Positive biological responses were also observed when adding ZnO [94] or SiAlON [95]. These findings indicate that the composition of Si_3_N_4_ could be further optimized for biological applications by exploring alternative additives to replace the conventional ones.

According to the literature, surface amines are effective in stimulating collagen, and in vitro testing performed using SAOS-2 osteosarcoma cells showed that the addition of Si_3_N_4_ to bioglass stimulated the production of more biological matrix compared to mineralized bone [35]. This effect has subsequently been confirmed also for bulk Si_3_N_4_ materials [36,37]. The results also indicate the upregulation of osteogenic transcription factors such as RUNX2, SP7, collagen type I, and osteocalcin.

Nitrogen and silicon elution, as observed by Pezzotti et al., are likely to occur on most Si_3_N_4_-based materials; however, a review of the literature suggests that these are not the key factors in regulating the biocompatibility. Spontaneously formed surface amines and osteo-inductive yttrium-rich phases are more likely to play the main role.

## 6. Anti-Pathogenic Mechanisms

Like previously discussed for bioactivity, the anti-pathogenic effects of Si_3_N_4_ have also been associated with the surface roughness, functional groups, release of antibacterial drugs, and presence of antibacterial secondary phases due to the introduction of additives. The bioactivity of Si_3_N_4_ can be explained via its surface chemistry, considering both the presence of surface amines and yttrium, while nitrogen elution is still the most likely mechanism responsible for the anti-pathogenic effects observed on most Si_3_N_4_ ceramics and Si_3_N_4_-based composites.

Unlike cells, bacteria are prokaryotic, meaning that they lack a complex internal structure like the nucleus and membrane-bound organelles found in eukaryotic human cells. This simplified structure makes them generally less equipped to handle the toxic effects of ammonia compared to human cells. On the other hand, many bacteria possess enzymes like urease that can detoxify ammonia by converting it into less harmful compounds. Certain human cells also have these enzymes, albeit to a lesser extent. A higher tolerance to ammonia/ammonium would also explain why, under normal testing circumstances, Si_3_N_4_ does not show the same cytotoxic effects as human cells, but the magnitude of this effect on bacteria should be evaluated case by case, as the tolerance varies greatly between species [96] and strains.

Gorth et al. compared the activity of several bacteria exposed to the surface of Si_3_N_4_ and other biomaterials [45]. Despite being the slowest to grow, *Enterococcus faecium,* a highly ammonia-tolerant bacteria often studied for its ammonia reduction potential [97], was the only strain to show some degree of resistance to the antibacterial activity of Si_3_N_4_. On the other hand, despite *S. epidermidis*, *S. aureus*, *E. coli*, and *P. aeruginosa* being all able to produce the enzyme, urease, its activity is generally limited compared to other urease-positive bacteria. Unfortunately, only a limited number of bacteria have been used for the in vitro testing of Si_3_N_4_ so far, and the list does not include other bacteria with strong urease activity, such as *Helicobacter pylori* [98], *Proteus mirabilis*, or *Staphylococcus saprophyticus*, meaning that a hypothetical correlation between urease activity and the antibacterial efficiency of Si_3_N_4_ cannot be demonstrated yet.

Some additional insights into the anti-pathogenic potential of ammonia released from Si_3_N_4_ can be deduced from the literature in vitro testing with *Candida* strains. Despite lacking the urease enzyme, *Candida albicans* uses urea amidolyase to hydrolyze urea and produce ammonia, which is its only source of nitrogen for the production of aminoacids [99]. Excesses of ammonium are then expelled from the cell and used for environmental pH modulation [100], but if the pH reaches a certain threshold, it might cause the inactivation of cell membrane enzymes resulting in a loss of biological activity [101]. Still, Si_3_N_4_ showed remarkable antifungal properties when tested against *C. albicans*, suggesting that ammonia release is not its main and/or only anti-pathogenic mechanism [48]. Tests have been conducted using amine-functionalized polymers [102,103], silica nanoparticles [104], and carbon nanotubes [105], showing exceptional fungicidal capabilities against *Candida albicans*.

As for the bioactive mechanisms discussed in the previous chapters, two possible pathways have emerged for the interpretation of the antibacterial capabilities of Si_3_N_4_, either due to ammonia/ammonium release or surface amines, with the latter being limited to the direct contact of the pathogen with the ceramic. One easy way to discriminate between the two mechanisms would be to perform a disk-diffusion antimicrobial susceptibility test on Agar plates and measure the inhibition area surrounding Si_3_N_4_: for the contact-based mechanism, the area would be negligible. Despite its simplicity and common use, such tests were never reported in the scientific literature. The authors are aware of such an investigation having been performed at least once between late 2014 and early 2015 with *Porphyromonas gingivalis*, *Treponema denticola*, and *Tannerella forsythia*, but the results were never published, as no antibacterial activity could be measured using this method on Si_3_N_4_ [106].

Another important remark from Gorth et al. is that the antibacterial activity of Si_3_N_4_ seems to be independent of the Gram classification [45]. This assertion has two potential consequences: Si_3_N_4_ might have a broader range of effectiveness compared to treatments that target specific cell wall structures and the Si_3_N_4_ antibacterial mechanism might not directly target the cell wall. Vancomycin, for example, is a glycopeptide antibiotic that works by interfering with the synthesis of the bacterial cell wall of Gram-positive bacteria, in particular targeting the peptidoglycan [107], but is ineffective against Gram-negative bacteria due to the presence of an outer membrane containing lipopolysaccharides.

Boschetto et al. studied the response of *E. coli* to Si_3_N_4_ surfaces by observing the metabolic alterations, in particular concerning the RNA and DNA structures within the bacterium. The authors reported “extensive disruption of the cell membrane”, suggesting that Si_3_N_4_ does attack cell walls directly in both Gram-positive and Gram-negative groups, but the claim is based on indirect spectroscopic alterations only and should be further validated through direct microscopic observation and biochemical analysis. The study concludes that “a high ammonium concentration outside the bacterial cell, usually referred to as “ammonium shock”, leads to lysis”, but it should be noted that this mechanism is not mentioned in the reference mentioned by the authors [108] or by the other recent scientific literature. Ammonium shock does cause the disruption of the metabolic processes and cellular functions, but this does not result in bacterial lysis [109,110,111]. Osmotic pressure, which was also observed by the authors [71], would be a more likely mechanism, that would also equally affect Gram-positive bacteria [112,113].

We can gain further insights into the antibacterial properties of Si_3_N_4_ by examining the extensive body of literature on AFM testing, which includes the studies on parameters like cell elasticity and adhesion performed with a Si_3_N_4_-coated tip [51,52,54,114]. The results suggest that the adhesion forces between Si_3_N_4_ and bacteria cell walls are present, but weak, while antibacterial effects or cell wall rupturing were never observed. Aucapina et al. observed that these forces increase over time, and eventually grow stronger than bacteria–bacteria interactions [115]. Bacteria adhesion to Si_3_N_4_ was shown to greatly vary even within the same bacteria species [55], and was ultimately postulated to be stronger for virulent strains, as a survival mechanism. Si_3_N_4_ was also shown to adhere to other micro-organisms’ membranes, such as the sporangia of the pathogenic oomycete *Plasmopara viticola*, leading to its death [56].

The role of additives in the anti-pathogenic mechanisms of Si_3_N_4_ has not been extensively explored in the literature. Du et al. noticed that the addition of rare earth oxides increases the antimicrobial properties [116], but the biocompatibility of such materials has yet to be investigated. Zeng et al. and Liu et al. observed an increased antibacterial efficiency when ZnO nanowires or whiskers are added to Si_3_N_4_ [94,117].

## 7. Si_3_N_4_ in Bioglasses

Due to its mechanical properties, Si_3_N_4_ has also been used as a strengthening agent for other bioactive ceramics creating composites. One of the first studies conducted by Santos et al. showed how adding a 30% proportion of Si_3_N_4_ in Bioglass^®^45S5 drastically improved the hardness and the fracture toughness with values complying with those usually reported for cortical bone. Consequently, in vitro bioactivity tests using SBF demonstrated how a thin layer of apatite was formed, suggesting how Si_3_N_4_ presence did not alter the bioactive effect of the bioglass [38]. An improvement of the mechanical properties was observed also by Amaral et al., who established the suitable conditions to fabricate almost fully dense silicon nitride biocomposites by hot pressing at 1350 °C–40 min–30 MPa [118]. A further confirmation of the improvement in the mechanical properties combined with a good bioactivity has been proved also by Frajkorova et al. Despite the composite being fabricated via sintering at 980 °C for 1 h in a nitrogen atmosphere, the specimen containing different concentrations of bioglass (10% and 30%, respectively) showed a better bioactivity via the immersion of composites in simulated body fluid for different time periods whereby the hydroxyapatite layer was developed on their surface [39]. The Si_3_N_4_ bulk used as a reference material exhibited no bioactivity, which clearly confirmed the positive effect of the bioglass addition. The first study analyzing the bioactivity of the composite Si_3_N_4_-bioglass through in vitro cells treatment was performed by Amaral et al. in 2002 [40]. Human bone marrow cells (MG63) were seeded on the composite specimen, and the performances were evaluated via proliferation (MTT assay), mineralization (SEM, ALP, and Alizarin Red assays), and protein level tests. The results showed a significant series of events associated with the rapid protein absorption of the surface, changes in the concentration of ionized Ca and P release in the medium, and high viability and proliferation of MG63. Also, the high ALP level and mineralized extracellular matrix produced by the cells confirmed the high bioactivity of the composites in bone formation. However, a Si_3_N_4_ bulk as reference was missing, and this did not provide information and data about the improvement associated with the use of Bioglass^®^.

Another study focused on improving the surface chemistry and topography of Zirconia-toughened alumina (ZTA) by using a powder mixture of silicon nitride and Bioglass^®^ was conducted by Marin et al. [35]. Initially the surface of the ZTA was treated via laser patterning, and the wells formed were filled with Bioglass^®^ or composites containing different concentrations of silicon nitride in Bioglass^®^ (5% and 10%, respectively). The results, after in vitro tests with the SaOS-2 cell line, showed how the presence of the bioactive powder and composites improved the cells’ adherence and bone formation on the surface. No differences in terms of extracellular matrix area coverage and volume were observed between the samples presenting Bioglass^®^ or composites, indicating how different amounts of silicon nitride did not affect the mineralization. Between the samples presenting fillers, only a variation in the mineral to matrix ratio by using FTIR was observed; by increasing the silicon nitride concentration, the ratio decreased, indicating a higher signal associated with Amide I, the organic component of the extracellular matrix. For the further confirmation of this effect provided by Si_3_N_4_, it would have been necessary to test the samples of ZTA presenting Si_3_N_4_ powder without Bioglass^®^ and also provide other tests straightly direct to analyze the collagen component (for example, the immunofluorescence staining of Col I) without basing the conclusion only on the FTIR spectra of a limited area of the specimens.

Summarizing the studies present in the literature, Si_3_N_4_ did not affect the bioactivity provided by Bioglass^®^, and concurrently improved the mechanical properties. However, despite the well-known antibacterial properties of Si_3_N_4_, no studies or data in the literature about the bacteriostatic behavior of composites such as Si_3_N_4_-Bioglass^®^ have been reported.

## 8. What Is Missing?

While other biomedical materials have advanced significantly through the use of additive manufacturing to better meet patient-specific needs, silicon nitride (Si_3_N_4_) is still lagging behind. Currently, Si_3_N_4_ implants are primarily produced using traditional ceramic processes, which involve powder compaction and high-temperature sintering. Though effective, these methods are costly, require extensive post-fabrication machining, and offer limited design flexibility [119]. Some progress has been made with additive manufacturing techniques like robocasting, which could enable custom Si_3_N_4_ implants with complex architectures and tailored porosity [120,121]. However, despite these advancements, Si_3_N_4_ has yet to fully embrace the additive techniques to the extent seen with other materials, leaving a gap in its application for patient-specific orthopedic solutions.

Si_3_N_4_ has shown potential in both spinal fusion and joint replacement applications, but its adoption in orthopedics remains limited due to several concerns. In spinal implants, while studies suggest that Si_3_N_4_ may offer advantages over other biomaterials, the variability in the clinical factors such as implant size, patient population, and biomaterial designs makes it difficult to generalize the findings. Moreover, the lack of randomized control trials and inconsistent definitions of subsidence complicate the assessment of Si_3_N_4_’s true efficacy [122]. In joint replacement, regulatory approval has not been granted, and concerns about its wear performance persist. Specifically, the surface oxide layer of Si_3_N_4_ may flake off, potentially leading to third-body wear, which could compromise the longevity of implants [72]. This uncertainty, coupled with the reliance on the integrity of the SiO_2_ film to maintain low friction, underscores the need for further investigation before Si_3_N_4_ can be widely adopted in orthopedic applications.

The biological applications of Si_3_N_4_ have been discussed numerous times in the literature, but despite the volume of scientific data and the general scientific consensus supporting the idea that Si_3_N_4_ bioceramics are biocompatible and bioactive, crucial information about the biological mechanisms involved is still missing.

The gap has been partially bridged by the integration of complementary techniques, in particular Raman spectroscopy. However, a potential over-reliance on Raman data has emerged in some fields. While spectroscopic techniques offer a powerful window into the chemical composition of biological samples, they inherently provide an indirect measure of the complex biological processes. Spectral data reflect the collective presence of various molecules, often lacking the specificity to distinguish between closely related compounds or functional groups. 

In the case of Si_3_N_4_, Raman spectroscopy has been often used as the main investigative tool, with little support from more conventional techniques. Unfortunately, so far, researchers have shown little interest in supporting or disproving these findings that are referenced based primarily on Raman data. This lack of investigation with complementary methods raises concerns about the generalizability and definitiveness of these conclusions.

Another critical aspect that has not been sufficiently discussed is the influence of the chemical composition. Commercial Si_3_N_4_ formulations often contain significant amounts of additives, some of which possess inherent bioactivity. However, this crucial factor is frequently disregarded, with some studies in the literature neglecting to even report the chemical composition of their Si_3_N_4_ samples. This lack of transparency hinders a comprehensive understanding of the observed effects and makes it difficult to distinguish between the contributions of Si_3_N_4_ itself and the potential bioactivity of its additives. 

In addition, most of the chemical and biological mechanisms reported in the literature appear to be very simplistic, based on circumstantial tests, and can be generated by the simple misinterpretation of spectra. Moreover, they often do not take into account the conditions that occur in vivo. In the absence of a scientific consensus on the surface chemistry, stoichiometry, and kinetics of the reactions of Si_3_N_4_ in biological or humid environments, it is impossible to achieve a comprehensive understanding of the biological mechanisms involved.

Another factor to consider is the fact that many studies tend to contradict each other, at least in what parameters determine the biocompatibility of silicon nitride. In recent years, there has been a growing body of literature focusing on the biological activity of silicon nitride (Si_3_N_4_) ceramics, highlighting their potential applications in biomedical fields. Several studies have provided evidence of silicon nitride’s favorable interactions with osteoblasts, promoting cell adhesion, proliferation, and mineralization. For instance, Neumann et al. found good osseointegration and minimal inflammatory reactions in vivo, supporting silicon nitride as a viable alternative to the established materials like alumina. However, contrasting findings from Griss et al. raised questions about its actual biocompatibility, with some studies reporting that Si_3_N_4_ exhibited inferior compatibility compared to other biomaterials.

Moreover, the differences in study outcomes can be attributed to various factors, including the specific processing methods employed, the composition of silicon nitride, and the presence of additives such as yttria (Y_2_O_3_). For instance, it has been suggested that the addition of yttria enhances the mechanical properties and bioactivity of silicon nitride ceramics, as noted by Bal et al., who found that yttria-containing Si_3_N_4_ exhibited superior cellular proliferation and mineralization compared to Si_3_N_4_ without additives. Conversely, other studies have indicated that the lack of standardization in sample preparation and testing conditions may contribute to the inconsistencies in the reported biocompatibility and bioactivity.

The antibacterial properties of silicon nitride also reflect a complex landscape of findings. Some researchers, such as Gorth et al., emphasized the material’s hydrophilicity and surface chemistry as critical factors for its antibacterial activity, while others have pointed to the role of nitrogen species released from the material, which may disrupt bacterial metabolism. Yet, the evidence remains inconclusive; the specific mechanisms underlying the antibacterial effects of silicon nitride are not fully understood, and there is a need for comparative studies that evaluate the efficacy of Si_3_N_4_ against a broader range of bacterial strains.

Additionally, many studies have focused on in vitro assays that, while informative, may not accurately reflect in vivo behavior. This variability underscores the necessity for more extensive investigations to validate the observed biological activities of silicon nitride ceramics. Collectively, these studies illuminate the complexities surrounding the biological responses to silicon nitride, emphasizing the importance of considering the interplay between material composition, surface characteristics, and the physiological context in which these ceramics are utilized. 

One last concern that must be raised is the low number of animal studies that have been performed on Si_3_N_4_ and published in the scientific literature. While Raman spectroscopy offers non-destructive insights into the molecular vibrations and material composition, its limitations, such as potential misinterpretations of spectra and difficulties in analyzing complex biomaterials, necessitate complementary techniques. In vitro evaluations play a critical role in the preliminary assessment of biomaterials, involving cultured cells to investigate the cytotoxicity, cell adhesion, and proliferation. For instance, cytotoxicity assays, such as MTT or Alamar Blue, measure the cell viability and metabolic activity, providing crucial insights into how materials affect the cellular health. 

However, in vitro evaluations are limited by their inability to fully replicate the complexity of living systems. Consequently, in vivo studies become indispensable. Such studies provide essential information on the tissue integration, systemic effects, and long-term biocompatibility, revealing how materials perform under physiological conditions. For example, in vivo assessments can highlight the formation of fibrous capsules around implants and the inflammatory responses that may occur, thereby offering insights into the biomaterial’s safety and efficacy. To gain a comprehensive understanding of the biological function, spectroscopic methods should be considered as complementary to the direct assessments of specific biochemical activities. Biological assays, for example, can quantify the enzymatic activity, gene expression, or protein–protein interactions, providing crucial information.

The few studies at our disposal are not sufficient to generate a solid statistical analysis. Furthermore, the existing studies present conflicting results, with some demonstrating very good bioactivity and others showing an almost complete lack thereof. This inconsistency in the findings further complicates the analysis and necessitates a more comprehensive research effort.

## 9. Conclusions

This review delved deeply into the intricate biological dynamics of Si_3_N_4_, scrutinizing its biocompatibility and antipathogenic potential across a spectrum of the literature spanning from works in the 1980s to contemporary studies. Despite a prevailing consensus on its biocompatibility and bioactivity, this analysis uncovered nuanced complexities. The shift in perspective from early studies, which dismissed any semblance of biocompatibility, to more recent findings demonstrating the cell growth on Si_3_N_4_ surfaces prompts the suspicion of a fundamental change in the ceramic’s chemistry over time. The production of advanced ceramics has evolved, and although the basic chemistry of the material has remained essentially unchanged, the effect of any additives has never been investigated. It is fair to assume that the chemical composition often takes precedence and that the role of additives like yttria remains underexplored yet is potentially influential in determining the Si_3_N_4_ biocompatibility. 

Moreover, the hypotheses surrounding Si_3_N_4_’s mechanisms, especially in the case of anti-pathogen behavior, are sometimes discordant. The rationale behind certain behaviors is occasionally attributed to the sample morphology, while at other times, it is linked to the chemistry of the material. Yet a definitive consensus and standardization of the sample preparation methods remain elusive and supported only by complementary techniques such as Raman spectroscopy, lacking cohesive validation through conventional methods. While in vitro findings hold promise, the scarcity and discordance of in vivo experiments underscore the imperative for collaborative, more comprehensive investigations.

## Figures and Tables

**Table 1:** List of major works examining Si_3_N_4_ biocompatibility in different forms and chemical compositions.

Authors	Composition/Producer	Ref	Bio Compatibility	Mechanism
Griss et al.	2.94 micron, meno 0.01 MgO, 0.073 CaO, 0.006 Fe_2_O_3_, 0.03 ZrO_2_	[1]	Bad	Unknown
Howlett et al.	Unknown	[8]	Good	Unknown
Griss et al.	2.94 micron, meno 0.01 MgO, 0.073 CaO, 0.006 Fe_2_O_3_, 0.03 ZrO_2_	[9,10]	Bad	Unknown
Sohrabi et al.	Unknown	[11]	Good	Unknown
Sohrabi et al.	Conducting Materials, Columbia, MD	[12]	Good	Roughness, chemistry
Neumann et al.	Mg 0.1, Al 1.7, O 4.3, N 55.6, Si 37.3, Y 1.0	[13]	Good	Unknown
Neumann et al.	Mg 0.1, Al 1.7, O 4.3, N 55.6, Si 37.3, Y 1.0	[14]	Good	Unknown
Neumann et al.	N 55.6, Mg 0.1, Al 1.7, Si 37.3, Y 1.0, O 4.3 N 50.8, Al 2.4, Si 40.7, Y 1.0, O 5.1 N 53.8, Mg 0.1, Al 1.1, Si 39.8, Y 1.5, O 3.8 N 48.9, Mg 0.5, Al 2.4, Si 41.5, Y 1.1, O 5.6 N 55.1, Mg 0.1, Al 1.7, Si 38.3, Y 0.9, O 4.0	[15]	Good	Unknown
Neumann et al.	Unknown	[16]	Good	Surface structures, chemistry
Bal et al.	N 53.30, Si 39.87, Y 1.1, O 4.1	[17]	Unknown	Unknown
Bal et al.	N 53.30, Si 39.87, Y 1.1, O 4.1	[18]	Unknown	Unknown
Bal et al.	N 53.30, Si 39.87, Y 1.1, O 4.1	[19]	Unknown	Unknown
Cappi et al.	10% Y_3_Al_5_O_12_	[20]	Good	Substrate chemistry and grain size but not roughness
Wang et al.	Si-O-N with high N/O ratio	[21]	Good	Substrate chemistry
Gustavsson et al.	Unknown	[22]	Good	Unknown
Yamamoto et al.	particles (700 µm)	[23]	Bad	Particle size and concentration
Bogner et al.	6 wt.% Y_2_O_3_ and 4 wt.% Al_2_O_3_	[24]	Bad	Unknown
Anderson et al.	Unknown	[25]	Good	Unknown
Webster et al.	Si_3_N_4_, Y_2_O_3_, Al_2_O_3_	[26]	Good	Unknown
Pezzotti et al.	Si_3_N_4_, Y_2_O_3_, Al_2_O_3_ As-fabricated Si 35.1 N 35.5 O 17.5 Al 2.1 Y 0.1 C 9.7 HF-etched Si 31.6 N 35.2 O 8.4 Al 0.9 Y 0.1 C 21.8 Oxidized Si 32.7 N 0.1 O 57.7 Al 2.9 Y 1.3 C 5.4 Thermal treatment in N2 Si 32.7 N 33.3 O 16.6 Al 5.1 Y 2.1 C 10.3	[27]	Good	Substrate chemistry
Sun et al.	Unknown	[28]	Bad	Unknown
Dion et al.	Unknown	[29]	Good	Unknown
Aydin et al.	Unknown	[30]	Bad	Particle size and concentration
Pezzotti et al.	Si_3_N_4_, Y_2_O_3_, Al_2_O_3_	[31]	Good	Substrate chemistry
Marin et al.	Si-rich Si_3_N_4_ N-rich	[32]	Good	Substrate chemistry
Marin et al.	6 wt.% Y_2_O_3_, 4 wt.% Al_2_O_3_, 90 wt.% Si_3_N_4_	[33]	Good	Surface roughness and chemistry
Zanocco et al.	6 wt.% Y_2_O_3_, 4 wt.% Al_2_O_3_, 90 wt.% Si_3_N_4_	[34]	Good	Surface roughness and chemistry
Marin et al.	Bioglass doped with 5 wt.% and 10 wt.% Si_3_N_4_	[35]	Good	Substrate chemistry
Ahuja et al.	6 wt.% Y_2_O_3_, 4 wt.% Al_2_O_3_, 90 wt.% Si_3_N_4_	[36]	Good	Substrate chemistry
Awad et al.	6 wt.% Y_2_O_3_, 4 wt.% Al_2_O_3_, 90 wt.% Si_3_N_4_	[37]	Good	Surface chemistry and wettability
Santos et al.	Unknown	[38]	Bad	Unknown
Frajkorová et al.	Si_3_N_4_-bioglass composite (100 wt.%, 90–10 wt.% and 70–30 wt.%)	[39]	Good	Bioglass addition
Amaral et al.	Si_3_N_4_-bioglass composite (70–30 wt.%)	[40]	Good	Unknown

**Table 2:** List of major works examining Si_3_N_4_ antipathogen activity in different forms and chemical compositions.

Authors	Composition/Producer	Ref	Antipathogen Activity	Mechanism
Bal et al.	Si_3_N_4_, Y_2_O_3_, Al_2_O_3_ As Fired and Polished surfaces	[45]	Staphylococcus Epidermidis (Good), Staphylococcus. Aureus (Good), Pseudomonas aeruginosa (Good), Escherichia coli (Good), Enterococcus (Good)	Hydrophilicity and surface chemistry
Webster et al.	Si_3_N_4_, Y_2_O_3_, Al_2_O_3_	[26]	Staphylococcus Epidermidis (Good),	Hydrophilicity and surface net charge
Pezzotti et al.	Si_3_N_4_, Y_2_O_3_, Al_2_O_3_	[46]	Porphyromonas gingivalis (Good)	Peroxynitrite formation
Bock et al.	6 wt.% Y_2_O_3_, 4 wt.% Al_2_O_3_, 90 wt.% Si_3_N_4_ As Fired N_2_-Annealed (SiYAlON excess on the surface) SiYAlON glazed Oxidized (Si-OH excess on the surface)	[47]	Staphylococcus epidermidis (Good)	Peroxynitrite formation
Pezzotti et al.	PMMA-βSi_3_N_4_ composite (6, 8, 10, 15, and 30 vol.%, no info on Si_3_N_4_ composition)	[48]	Candida albicans (Good)	Chemical and osmotic stress
Pezzotti et al.	6 wt.% Y_2_O_3_, 4 wt.% Al_2_O_3_, 90 wt.% Si_3_N_4_	[49]	Human herpesvirus 1 (Good)	Peroxynitrite formation
Ishikawa et al.	6 wt.% Y_2_O_3_, 4 wt.% Al_2_O_3_, 90 wt.% Si_3_N_4_	[50]	Staphylococcus. Aureus (Good),	Peroxynitrite formation and surface morphology
Fang et al.	Unknown	[51]	Sulfate-reducing bacteria (Bad)	Weak adhesion
Yao et al.	Unknown	[52]	Pseudomonas aeruginosa (Bad) Enterococcus hirae (Bad)	Weak adhesion
Pinar Gordesli et al.	Unknown	[53]	Listeria monocytogenes (Bad)	Weak adhesion
Boonaert et al.	Unknown	[54]	Phanerochaete chrysosporium (Bad) Lactococcus lactis (Bad)	Weak adhesion
Park et al.	Unknown	[55]	Listeria monocytogenes (Bad)	Unknown
Pezzotti et al.	6 wt.% Y_2_O_3_, 4 wt.% Al_2_O_3_, 90 wt.% Si_3_N_4_	[56]	Plasmopara viticola (Good)	Peroxynitrite formation

## Data Availability

Data are contained within the article.
